# *Didymocarpus phuquocensis*, a new species of Gesneriaceae from Phu Quoc Island, South-western Vietnam

**DOI:** 10.3897/phytokeys.159.47442

**Published:** 2020-09-04

**Authors:** Thi-Lien Tran, Ngoc-Sam Ly, Minh-Ngoc Tran, Xuan-Truong Nguyen, Ngoc-Giang Cao, Hong-Dung Pham

**Affiliations:** 1 National Institute of Medicinal Materials, 3B Quang Trung, Hoang Kiem District, Ha Noi,Vietnam National Institute of Medicinal Materials Ha Noi Vietnam; 2 Institute of Tropical Biology, VAST, 85 Tran Quoc Toan road, District 3, Ho Chi Minh City, Vietnam Institute of Tropical Biology Ho Chi Minh City Vietnam; 3 Graduate University of Science and Technology, VAST, 18, Hoang Quoc Viet, Cau Giay District, Ha Noi, Vietnam Vietnam Graduate University of Science and Technology Ha Noi Vietnam; 4 Phu Quoc National Park, 1 Nguyen Chi Thanh, Duong Dong Town, Phu Quoc District, Kien Giang Province, Vietnam Phu Quoc National Park Duong Dong Town Vietnam

**Keywords:** *
Didymocarpus
*, Kien Giang, new species, taxonomy, Vietnam

## Abstract

A new species of Gesneriaceae, *Didymocarpus phuquocensis*, is described and illustrated from Phu Quoc National Park, Kien Giang Province, South-western Vietnam. It is most similar to *D. pulcher*, *D. hookeri* and *D. punduanus* in having 3-verticillate petiolate leaves, morphologically similar calyx, corolla, stamens, pistil and fruit, but differs from all in the glandular-pubescent stems, petioles and leaf blades, 1(–2)-flowered cymes, longer corolla and fruit and longer and densely glandular-puberulent ovary. Data on distribution, ecology, phenology and provisional conservation assessment of the new species are given along with an illustration and a colour plate.

## Introduction

The genus *Didymocarpus* Wallich was established in 1819, based on the species *D. primulifolius* D.Don from Nepal. The genus has previously been considered to comprise about 180 species distributed in tropical Asia with a few scattered in Africa and Australia (Wallich 1819; Don 1825; Wang et al. 1998; Weber and Burtt 1998; Weber et al. 2000). The taxonomic delimitation of the genus *Didymocarpus* has varied considerably over time (e.g. Burtt 1998; Weber et al. 2000; Möller et al. 2011; Möller and Clark 2013; Li et al. 2015). By combining molecular phylogenetic data and morphological revision of *Didymocarpus*, recent studies have remodelled and reduced this number (Vitek et al. 2000; Weber et al. 2000, 2011a, b; Möller et al. 2011; Li et l. 2015; Möller et al. 2017), with some species being placed in other genera, particularly to *Henckelia* Spreng. (ca. 60 species (Weber et al. 2011b, Middleton et al. 2013)), *Petrocodon* Hance (more than 20 species (Weber et al. 2011a)) and *Tribounia* D.J.Middleton (2 species (Middleton and Mӧller 2012)). The genus, as currently recognised, has around 98 species (Möller et al. 2017). Species of *Didymocarpus* are distinguished from other genera of Gesneriaceae by having: lithophyte perennial habit, ovate to ovate-cordate lamina which is mostly glandular-hairy, tubular corolla with an oblique limb (rarely trumpet-shaped or bell-shaped), two fertile stamens, three staminodes, a capitate stigma, an orthocarpic ovary and a bivalve capsule which dehisces loculicidally (Weber et al. 2000). As currently circumscribed, *Didymocarpus* ranges from northwest India, eastwards through Nepal, Bhutan, northeast India, Burma (Myanmar), southern China, Vietnam, Laos, Cambodia, Thailand and the Malay Peninsula, with the highest species diversity being found in China and Thailand (Weber and Burtt 1998; Weber et al. 2000; Nangngam and Maxwell 2013; Nangngam and Middleton 2014; Möller et al. 2016). In Vietnam, five species of *Didymocarpus* have been reported, namely *D. bonii* Pellegr., *D. kerrii* Craib, *D. poilanei* Pellegr., *D. pulcher* C.B.Clarke and *D. purpureobracteatus* W.W.Sm. (Pellegrin 1930; Pham 2000; Phuong and Xuyen 2012; Phuong et al. 2014), but *D. bonii* Pellegr. [= *Calcareoboea bonii* (Pellegr.) Burtt] has been recognised as *Petrocodon bonii* (Pellegr.) A.Weber & Mich.Möller (Weber et al. 2011a). Recently, *Didymocarpus puhoatensis* X.Hong & F.Wen was described from Central Vietnam (Hong et al. 2018).

During medicinal plant investigations in Phu Quoc National Park (NP), Kien Giang Province, south-western Vietnam, several interesting plants of a small species of Gesneriaceae were collected by the authors in 2018–2019. The flowers of these plants have a capitate stigma and other features characterising this plant as *Didymocarpus* (Wang et al. 1998, Weber et al. 2000). A critical examination of living flowers, herbarium specimens of these plants and comparison with type material and protologues of all closely-related species in Vietnam and neighbouring countries (e.g. Clarke 1874, 1883, 1884; Wallich 1829; Wang et al. 1998; Pham 2000; Nangngam and Maxwell 2013; Phuong et al. 2014; Nangngam and Middleton 2014; Sinha and Datta 2016; Roy 2017), suggested that these specimens were different from the other known *Didymocarpus* species. These plants with 3-verticillate, petiolate leaves, campanulate calyx, funnelform corolla found in Phu Quoc NP show similarities in these characters with *D. pulcher*, *D. hookeri* C.B.Clarke and *D. punduanus* Wall. ex R.Br. However, it shows significant differences in its vegetative and floral structures (see Table 1) and we describe it here as a species new to science (see also Taxonomic Notes).

**Table 1.** Morphological comparison of *D. puhoatensis* with its most closely-related taxa (based on Wallich 1829; Clarke 1874, 1883, 1484; Sinha and Datta 2016; Roy 2017).

Characters	*D. phuquocensis*	*D. pulcher*	*D. hookeri*	*D. punduanus*
**Plant height**	(8–)10–19 cm	ca. 30.5 cm	ca. 40 cm	20.3–25.4 cm
**Stem**	glandular-pubescent	puberulous, eglandular	villous, eglandular	pubescent, eglandular
**Petiole**	glandular-pubescent	puberulous, eglandular	villous, eglandular	pubescent, eglandular
**Leaves**	3-verticillate petiolate leaves, terminal whorl of smaller sessile leaves	3–4- verticillate petiolate leaves, terminal whorl of smaller sessile or subsessile leaves	usually 3–4- verticillate petiolate leaves, uppermost leaves sessile	3- verticillate petiolate leaves, 2 sessile leaves at the apex
**Leaf blade**				
Margins	serrate	crenulate to serrate	crenulate	shallowly crenulate
Base	attenuate or cuneate	auriculate cordate	cordate	cordate
Apex	attenuate to acute	short acute	subobtuse	subobtuse
Indumentum	glandular-pubescent	pubescent, eglandular	somewhat villous eglandular	puberulous, eglandular
**Inflorescence**	axillary or terminal 1(–2)-flowered cyme	axial and terminal many-flowered cyme	terminal many-flowered cyme	terminal many-flowered cyme
**Bracts**	oblong-lanceolate, abaxially densely multicellular glandular-pubescent	rounded/suborbic-ular, sparsely viscous pilose	rounded, glabrescent	ovate, nearly glabrous
**Corolla**	4.8–5.3 cm long, light purple, glabrous	2.5–3 cm, violet-purple, glabrous or outside sparsely multicellular-villous	ca. 1.4 cm long, pale yellow with rose marks or nearly white, outside pilose	ca. 2.2 cm long, purple, nearly glabrous
**Ovary**	ca. 3 cm long, densely multicellular glandular-puberulent	1.1–1.8 cm, glabrous	1.1–1.7 cm long, pubescent	0.7–1.3 cm long, glabrous
**Style**	sparsely glandular-puberulent	glabrous	glabrous	glabrous
**Capsule**	4.4–5.5 cm long	ca. 4 cm long	1.3–3.5 cm long	ca. 4 cm long

## Materials and methods

The descriptions are mainly based on measurements from flowering material of living plants in the field, supplemented by measurements from herbarium specimens. Type specimens of the most closely-related species were examined from the herbaria material from the following herbaria: E, K, HN, IBK, P, VNM, VNMN and W (herbarium codes follow Thiers (2020)), as well as digitised specimen images of *Didymocarpus* species also being accessed from botanical websites (e.g. https://science.mnhn.fr/, http://www.cvh.org.cn/, https://plants.jstor.org/). All morphological characters were studied under a dissection microscope and are described using the general terminology and standard work of Wang et al. (1998) and Harris and Harris (2001). A distribution map was created using SimpleMappr (http://www.simplemappr.net/) (Shorthouse 2010). Conservation status was assessed using the IUCN Red List Categories and Criteria version 3.1 (IUCN 2019) and inferring from the GeoCAT website (http://geocat.kew.org/editor) (Bachman et al. 2011).

## Taxonomic treatment

### Didymocarpus phuquocensis

Taxon classificationPlantaeLamialesGesneriaceae

N.S.Lý, T.L.Tran & N.G.Cao sp. nov.

F638E108-BD55-54EF-9276-057320CEAFE6

urn:lsid:ipni.org:names:77211380-1

Figures 1 , 2

#### Diagnosis.

*Didymocarpus phuquocensis* is most similar morphologically to *D. pulcher, D. hookeri* and *D. punduanus* in the 3-verticilate, petiolate leaves, the morphologically-similar calyx, corolla, stamens, pistil and fruit, but differs from all in the glandular-pubescent stems and petioles (vs. puberulous, villous, pubescent and eglandular of the latter three, respectively), glandular-pubescent leaf blades (vs. pubescent, somewhat villous, puberulous and eglandular, respectively), 1(–2)-flowered cymes (vs. many-flowered cymes of the latter three), longer corolla 4.8–5.3 cm long (vs. 2.5–3 cm in *D. pulcher*, ca. 1.4 cm in *D. hookeri* and ca. 2.2 cm in *D. punduanus*), longer and densely multicellular glandular-puberulent ovary ca. 3 cm long (vs. 1.1–1.8 cm and glabrous in *D. pulcher*, 1.1–1.7 cm and pubescent in *D. hookeri* and 0.7–1.3 cm and glabrous in *D. punduanus*) and longer fruits 4.4–5.5 cm long (vs. ca. 4 cm long in *D. pulcher*, 1.3–3.5 cm long of *D. hookeri* and ca. 4 cm long in *D. punduanus*).

#### Type.

Vietnam. Kien Giang Province: Phu Quoc District, Phu Quoc NP, Suoi Mo, 18 September 2018, 10°14'40.90"N, 104°2'14.15"E, 82 m elev., *Lý Ngọc Sâm*, *Cao Ngọc Giang*, *Nguyễn Thị Liên*, *Ngô Minh Huyền*, *Hùng, Hà Văn Long*, *TNB-305* (Holotype: VNM, isotype: P, NIMM).

**Figure 1.**
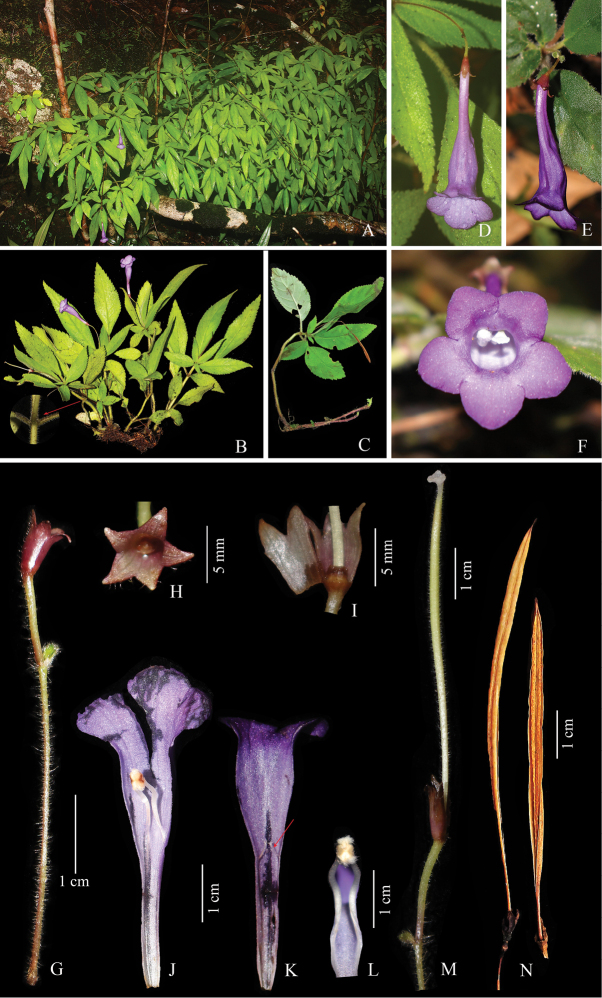
*Didymocarpus phuquocensis***A** flowering plants in natural habit **B** flowering plants showing 3-verticillate leaves (red arrow) **C** mature plant with dried fruit and new stems **D** flower (top view) **E** flower (side view) **F** flower (front view) **G** inflorescence with peduncle, bract and calyx **H** calyx (top view) **I** longitudinal section of calyx showing disc **J** longitudinal section of corolla showing anterior (lower) lip with fertile stamens **K** longitudinal section of corolla showing posterior (upper) lips and staminodes (red arrow) **L** close-up of fertile stamens **M** ovary and calyx **N** dried fruits. The coloured plate prepared by Ngọc-Sâm Lý.

#### Description.

Deciduous, perennial, epilithic herb, (8–)10–19 cm tall, stems 2.5–3.5 mm in diameter. ***Dry season***: new vegetative buds produced from the rhizome which then develops during the rainy season. ***Rainy season***: stem erect, (3–)4 nodes, pale greenish, densely white multicellular glandular-pubescent; the longest node separated from the base of stem 5.7–12.2 cm long, the medium nodes at the middle stem 1.5–4.5 cm long, the shortest nodes very shortly distanced (0.2–0.5 cm long) at the apex. **Leaves** 3- verticillate, petiolate in the 2^nd^ and 3^rd^ whorls, other whorls with smaller and sessile or subsessile leaves; blades coriaceous, adaxially light greenish, abaxially whitish-green, asymmetrically narrowly elliptic to elliptic-ovate, the largest ones 7.2–10 × 2.4–3.7, the smaller ones 1–4.6 × 0.5–2.1 cm, adaxially densely white multicellular glandular-pubescent, abaxially sparsely white multicellular glandular-pubescent, apex attenuate to acute, base lightly oblique, attenuate to cuneate, margin serrate; venation pinnate, with 5–6 of ascending secondary veins on each side of midrib, somewhat opposite, adaxially obscure, abaxially prominent, densely covered with indumentum as the stem; petioles terete, unequal in length, 0.5–3.2 cm long [the longest ones 3–3.2 cm, the shortest ones 0.5–1 cm], 2–2.5 mm in diam., whitish-green, sometimes tinted greenish-purple above, with indumentum as the stem. **Inflorescences** terminal or subterminal, cyme 1(–2) flowered, pendent; **peduncle** slender, (1.3–)3.1–4.5 cm long, ca. 0.5 mm in diam., tinted reddish-green, covered with white multicellular glandular and glandular-pubescent; **pedicels** 5–11 mm long, ca. 0.7 mm in diam., pale green, with indumentum as the peduncle, but more sparse; **bracts** paired; lanceolate to oblong-lanceolate, 4–5 × 1–1.5 mm, apex round to acute, margin entire, green, adaxially sparsely multicellular glandular-pubescent, abaxially densely white multicellular glandular-pubescent. **Calyx** campanulate, 5–6.5 mm long, dull reddish, outside sparsely multicellular glandular-puberulent; tube 3–4 mm long, 2–2.5 mm in diam.; lobes triangular, (sub)equal, 5-lobed, symmetrical, 2–2.5 mm long, ca. 1.5–1.8 mm wide at base, apices acute. **Corolla** funnelform, 4.8–5.3 cm long, glabrous, light purple, paler at base; tube 3.8–4.1 cm long, base narrow, 2–3 mm in diameter, widening abruptly at 1.9–2.1 cm from the base, widest at throat, 1–1.1 cm in diam.; lobes (sub)orbicular; anterior (lower or abaxial) lip 3-lobed, unequal, the middle one 5–7 × 7–10 mm, the lateral ones 5–7 × 4.5–5 mm, apices rounded; posterior (upper or adaxial) lip 2-lobed, slightly equal, 5–6 × 7.5–8.5 mm, apices rounded. **Stamens** 2, inserted at 2.2–2.4 cm above the base of the corolla; filaments slender, white, glabrous, 7–8 mm long, glandular-puberulent on the connective; anthers brownish, oblong, 2–2.2 × ca. 1 mm, tips and bases rounded, white-bearded; **staminodes** 3, inserted ca. 3 mm below the stamens, reduced to filaments, equal in length, 2.5–3.5 mm long, glabrous, tips with few glandular-puberulent. **Disc** cupular, ca. 1 mm high, margin irregular sinuate. **Pistil** ca. 3.2 cm long; **ovary** cylindrical, greenish with white towards the base, ca. 3 cm long, 1–1.5 mm in diam., densely glandular-puberulent; **style** continuous with the top of the ovary, ca. 2 × 1 mm, whitish, sparsely glandular-puberulent; **stigma** irregular capitate, ca. 1 × 1 mm, concave, white, papillose. **Capsules** straight to slightly curved, linear, glabrous, 2-valved, loculicidal dehiscent, 4.4–55 cm long, 1–1.5 mm in diam., turning brown when ripe, calyx and style persistent. **Pollen** and **Seeds** not studied.

#### Distribution and habitat.

**Figure 2.**
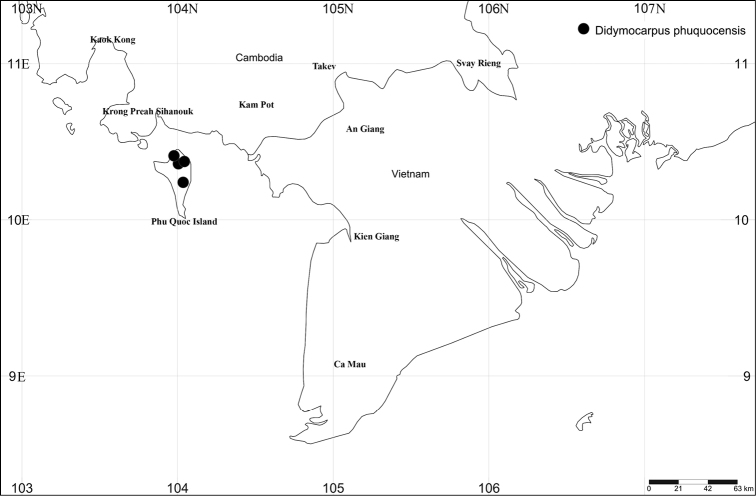
Distribution map of *Didymocarpus phuquocensis* in Phu Quoc National Park, Phu Quoc Island, Kien Giang Province.

This species grows in moist places and shaded areas in primary tropical evergreen forests, on granite bedrock along streams or on moist and mossy cliffs in Phu Quoc NP, at 80–530 m elev. (Fig. 2).

#### Phenology.

Flowering and fruiting from May to October.

#### Etymology.

The specific epithet “*phuquocensis*” was named after the type locality.

#### Provisional conservation status.

At present, four relatively-large subpopulations consisting of about 2000 mature individuals/mature clumps have been observed in Phu Quoc NP. The extent of occurrence (EOO) and the area of occupancy (AOO) were estimated using the web Geospatial Conservation Assessment Tool or GeoCAT (Bachman et al. 2011) and the auto-value cells width 2 km were calculated to be 53.7 km^2^ and 16 km^2^, respectively. These two values meet the criteria B1 (EOO < 100 km^2^) for Critically Endangered and B2 (AOO < 500 km^2^) for Endangered, following the IUCN Red List Categories and Criteria Version 3.1 (IUCN 2019). Although the known habitat of the new species is protected as part of the National Park, its habitat is fragmented and still faces some risk due to loss of the habitat within some parts of its range (in particular the clearing of forest land for agricultural fields and building of numerous roads and tourism areas). Based on the IUCN Red List Criteria (IUCN 2019), we therefore provisionally assess this species as Endangered (EN B2ab(iii), C).

#### Other specimens examined

**(*Paratypes*).** Vietnam. Kien Giang Province: Phu Quoc Island, Phu Quoc NP, K7 peak, 10°21'53.42"N, 104°0'31.22"E, 356 m elev., 21 May 2019, *Lý Ngọc Sâm*, *Hà Văn Long*, *TNB-430* (VNM); the same locality, Nui Chua peak, 10°22'40.09"N, 104°2'6.24"E, 532 m elev., 22 July 2019, *Cao Ngọc Giang*, *Ngô Minh Huyền*, *Hà Văn Long*, *TNB-502* (VNM); the same locality, Ham Rong Mount, 10°24'6.34"N, 103°58'6.47"E, 351 m elev., 10 July 2019, *Cao Ngọc Giang*, *Ngô Minh Huyền*, *Hà Văn Long*, *TNB-508* (VNM).

#### Vernacular name.

Vietnamese language: Song bế phú quốc.

#### Taxonomic notes.

Morphologically, the 3-verticillate petiolate leaves of *D. phuquocensis* are shared with several species of *Didymocarpus*, such as *D. insulsus* Craib (north-eastern, Thailand), *D. tristis* Craib (Chanthaburi Province, south-eastern Thailand), *D. dongrakensis* B.L.Burtt (northeast Thailand) *D. newmanii* B.L.Burtt (Chanthaburi Province, south-eastern Thailand), *D. pulcher* (from India, Buhtan, Nepal, China to Vietnam), *D. hookeri* (Assam, Arunachal Pradesh, Meghalaya and Sikkim, India), *D. punduanus* (Assam, Meghalaya and Nagaland, India) (Wang et al. 1998; Nangngam and Maxwell 2013; Phuong et al. 2014; Sinha and Datta 2016; Roy 2017). Of these, the campanulate calyx of the new species is similar to *D. pulcher*, *D. hookeri* and *D. punduanus*, but distinguished from *D. insulsus*, *D. newmanii* and *D. tristis* which have the calyx 5-lobed to the base. *Didymocarpus phuquocensis* is most similar to *D. pulcher*, *D. hookeri* and *D. punduanus* in the morphological characters of 3-verticillate petiole leaves, the same shape of calyx, corolla, stamens, pistil and fruit. The major differences between the new species and the three latter are outlined above in the diagnosis. Moreover, the shorter plant height ((8–)10–19 cm), the narrowly elliptic to elliptic-ovate leaf blades that have serrate leaf margins, attenuate to cuneate leaf base and attenuate to acute leaf apex, the oblong-lanceolate bracts being abaxially densely multicellular glandular-pubescent, the light purple corolla and sparsely glandular-puberulent styles of *D. phuquocensis* also distinguish it from *D. pulcher*, *D. hookeri* and *D. punduanus*. A detailed morphological comparison between *D. phuquocensis*, *D. pulcher*, *D. hookeri* and *D. punduanus* is provided in Table 1.

## Supplementary Material

XML Treatment for Didymocarpus phuquocensis
